# Is there a frontier in sensitivity with Lossy mode resonance (LMR) based refractometers?

**DOI:** 10.1038/s41598-017-11145-9

**Published:** 2017-08-31

**Authors:** Aritz Ozcariz, Carlos R. Zamarreño, Pablo Zubiate, Francisco J. Arregui

**Affiliations:** 1 Department of Electrical and Electronic Engineering, Public University of Navarre, 31006 Pamplona, Spain; 2 Institute of Smart Cities, Public University of Navarre, 31006 Pamplona, Spain

## Abstract

A tin dioxide thin layer has been studied in order to improve the sensitivity of lossy mode resonances (LMR) based sensors. The effects of the thin film thickness and the polarization of light in a SnO_2_ coated D-shaped single mode optical fiber have been evaluated. The optimization of such parameters in the fabrication of refractometers have led to an unprecedented sensitivity of over one million nanometers per refractive index unit (RIU), which means a sensitivity below 10^−9^ RIU with a pm resolution detector. This achievement is a milestone for the development of new high sensitivity devices and opens the door to new industrial applications, such as gear oil degradation, or biomedical devices where previous devices could not provide enough sensitivity.

## Introduction

Optical fiber was primarily designed as a waveguide of great capabilities such as extremely low loss, great bandwidth, and isolation to external noise and signals. In spite of that, several interrogation techniques and fiber manufacturing procedures have been developed for the use of optical fiber devices as sensors. These configurations include fiber Bragg gratings (FBG)^1^, long-period fiber grating (LPG)^2^, interferometers^1^, photonic crystal fibers (PCF)^3^, hollow-core fibers (HCF)^4^, single-multi-single (SMS) structures^5^, etc.

A different approach is based on electromagnetic resonances induced in the optical fiber. Here, when an uncladded fiber is coated with certain materials, part of the light can be propagated along this film, generating a resonance. Two major kinds of resonances have been mainly described in literature attending to the properties of the thin film: surface plasmon resonances (SPR) and lossy mode resonances (LMR)^6^. Both kind of resonances can be generated on a coated optical fiber, but the permittivity of the coating determines the kind of resonance induced and the position within the optical spectrum among other things^7^. In general, a SPR can be obtained when the real part of the complex permittivity of the coating is negative and greater in magnitude than its imaginary part and the real part of the external medium permittivity. The same conditions apply for the LMR generation, as long as the real part of the coating permittivity is positive^6^. These conditions imply that only conductive materials (metals and semiconductors) can be used for SPR generation, whereas a wider range of materials such as dielectrics, semiconductors or polymers can be used for the fabrication LMR supporting thin films. The development of these resonance-based optical fiber sensors enable to obtain very high sensitivity devices and has created a new range of possibilities in the field of sensing. In particular, SPR devices are the gold standard in the industry of biomedical sensing. SPR optical fiber configuration offers several benefits such as the size reduction, cost and simplicity in contrast to the typically represented SPR Kretschmann configuration (based on a coated bulk prism).

Polarization control is one important aspect to consider in the fabrication of resonance based optical fiber sensors. While SPR are only generated in the TM mode, the LMR occur in both TM and TE (Transverse Electric) Mode^8, 9^. Besides being one of the main differences of LMR that greatly simplifies the setup needed for its use, it has also additional advantages. LMR generated in each mode (TM and TE) are not obtained exactly at the same wavelength and, in fact, they have a different sensitivity, which might causes the resonances to widen and distort their shape^9^. However, this can be exploited by isolating the modes (LMR_TE_ and LMR_TM_) propagated through the fiber obtaining resonances with a better shape and increased sensitivity. A special type of fiber, D-Shape fiber, enables to observe this phenomenon using TiO_2_ coated D-shape fiber^9^. In this way, both polarization modes can be studied separately. A similar approach had been previously reported using a zinc oxide coated D-Shape fiber^10^, although the resonances observed in that research had not been considered to be LMR at that point.

The flexibility of the use of many different materials and fabrication techniques is another advantage of LMR based devices. LMR based optical fiber sensors have been obtained using indium tin oxide (ITO)^11–13^, Titanium dioxide^14^ or zinc oxide^15^ and polymeric^16^ coatings fabricated by means of different techniques that include layer-by-layer^16^, sputtering^11, 12^, thermal evaporation^15^, dipping technics^15, 17^, etc. Theory states that fabricating a thin-film with the optimal optical properties can maximize the sensitivity of the LMR^7^. The most important property to consider is the complex refractive index. The real part, n, should be high, while the imaginary part, k (also known as coefficient of extinction) should be close (but not equal) to zero. When those conditions are matched, a resonance will be generated at a given wavelength, depending on the thickness of the coating and the refractive index of the surrounding media (SMRI). Amongst the previously mentioned parameters for LMR generation, tin dioxide (SnO_2_) exhibits the most appropriate properties, in terms of refractive index, in order to maximize sensitivity of a LMR based sensor. Apart from its optical properties, SnO_2_ is a relatively cheap and available material, easy to deposit through different techniques such as DC sputtering^11^, spray^18^, sol-gel^19^, and is widely studied material for sensing applications^20^.

The aim of the research presented in this paper is to experimentally achieve an unprecedented sensitivity in LMR based optical fiber devices. In order to do so, the obstacle of controlling with precision the thickness of the deposited coating had to be solved, since a small variation affects such sensitive resonances. To our best knowledge, no refractometer based on LMR has been reported experimentally in the literature with such a high sensitivity as the one presented here.

## Results and Discussion

### Thin film and resonance characterization

The location of the LMR in the spectrum depends on the thickness of the SnO_2_ coating. A thicker film will produce a resonance at a longer wavelength. Therefore, it is important to study the location of the LMR depending on the thickness of the coating. In order to do so, a deposition cycle was performed for 50 minutes while the transmitted optical power at two different wavelengths (1310 and 1550 nm) was monitored. Although the fiber obtained after this experiment is not useful for sensitivity measurement purposes the experiment enables to determine the location of the LMR as a function of the thickness of the coating. During the deposition, a piece of silicon wafer was placed next to the fiber to act as a probe. A posterior SEM measurement of this and other samples of 2, 3 and 4 minutes deposition time, allowed to determine an average deposition rate of 0.793 nm/s (Supplementary Table S1, and Supplementary Figures S1–S15).

**Figure 1:**
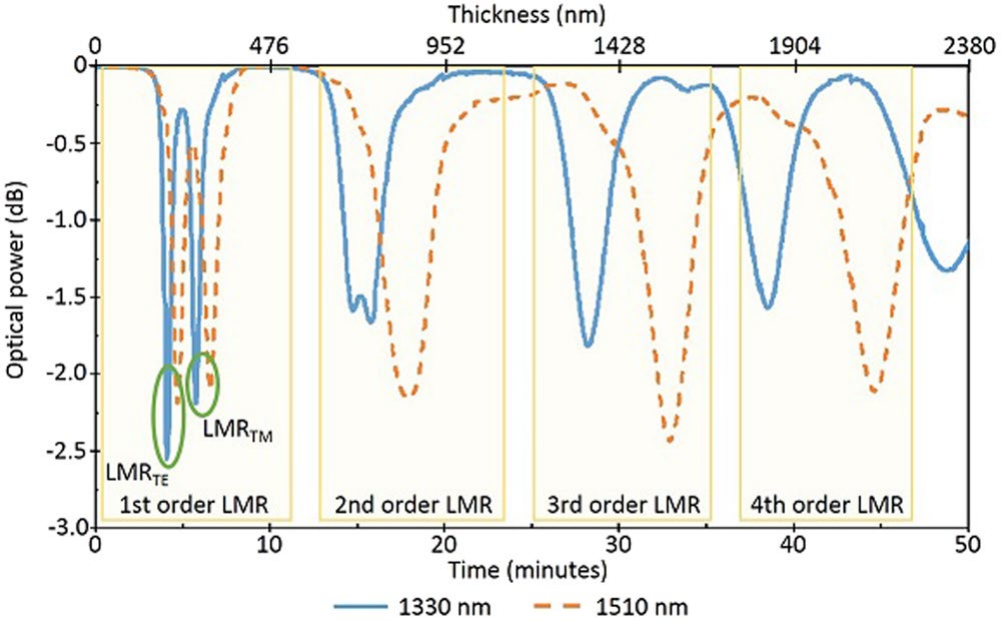
Output optical power evolution as the thickness of the thin film increases. The bottom axis shows the deposition time while the top axis represent the expected coating thickness at that moment. Several resonances can be distinguished. In the first order LMR observed, two components can be appreciated, corresponding each to a different mode.

The results are represented in Fig. 1 where the transmitted optical power at 1310 nm and 1550 nm varies with time, when the thickness of the thin film increases. Several attenuations can be observed in the graphic, first at 1310 nm and then at 1550 nm. These attenuations are associated to a coupling between the waveguide mode and a particular lossy mode in the SnO_2_ coating that takes place at a particular wavelength depending on the thickness of the SnO_2_ coating. In other words, these attenuations correspond to several LMR observed at the two mentioned wavelengths as the thickness of the deposited film is increased. The LMR are generated at lower resonances first and, as the thickness of the coating increases, they shift to longer wavelengths. It is important to note that in this setup the light is depolarized and, as a result, the sum of the components of both polarizations will be observed. Due to this, in the first order LMR the two components corresponding to the TE and TM modes can be easily differentiated whereas in the rest they are closer and we only observe the sum of both components.

All the resonances have the same behaviour, but their sensitivity varies. Those of lower order have proven to be the ones with the highest sensitivity^7^, being the sensitivity of the resonance generated in the TE mode (LMR_TE_) slightly different from the one of the resonance generated in the TM mode (LMR_TM_). In Fig. 1, this effect can be clearly observed. As the order of the resonance increases, the increment in thickness needed for the shift from 1330 to 1510 nm is bigger. This means that its sensitivity to the thickness of the thin film decreases.

Another aspect to consider is the fact that these are the positions of the resonances when the fiber is surrounded by air (or a media where the refractive index is close to 1). When the fiber is submerged in a water-like media with higher refractive index, the LMR shifts to higher wavelengths (and will go out of the spectral range to monitor if the resonance is too sensitive). Therefore, as our spectral window is limited, it must be taken into account that the resonances observed when the fiber is submerged in water or glycerin solutions, are not yet observed when the fiber is surrounded by air. This is an added difficulty to tune the LMR in our spectral window when submerged in the glycerin solutions. Once a D-shape fiber has been coated with a SnO_2_ film, it must be submerged in several solutions with refractive index from 1.3210 (water at 1310 nm) to 1.4493 (water/glycerin 90%) to observe the exact location of each resonance for that thickness.

### Refractometer characterization

Once the control of the thickness of the SnO_2_ film is well established, D-Shaped fibers were coated with a layer that generates resonances within the working wavelength range of the equipment (1150–1650 nm). Such coating has a thickness of only 14 nm (See Supplementary fig. S16). Thinner coatings allow to observe resonances for higher values of surrounding media refractive index (SMRI). However, there are two main limitations. On the one side, if the coating is too thin, it might lose homogeneity, leading to distorted resonances. On the other side, as the SMRI increases, it approaches the value of the refractive index of the optical fiber, and induces an absorption increase that masks the resonances.

These fibers will be installed in a setup that allows to record the absorption spectrum while the fiber is sequentially submerged in glycerin solutions of different refractive index, in order to characterize the device as a refractometer. Since the refractive index of the solutions is dependent of the temperature, all measurements were performed at a constant room temperature of 25 °C. In this case it is required the utilization of a polarization controller in order to isolate each mode (TE and TM) and observe each resonance (LMR_TE_ and LMR_TM_) separately. The separation of each mode makes possible to study resonances much narrower and with better figure of merit than using depolarized light. From previous studies^21^, the first LMR (the one observed for lower refractive index) usually appears in the TE mode and is followed closely by the one induced in the TM mode. It has been previously reported that higher sensitivities are obtained at high refractive index solution^7^. This require a precise control of the thickness of the coating in order to position the resonance within the working wavelength range as well as an accurate adjustment of the refractive index of the solutions because a very small change or deviation in any of them could shift the resonance out of the optical window.

**Figure 2:**
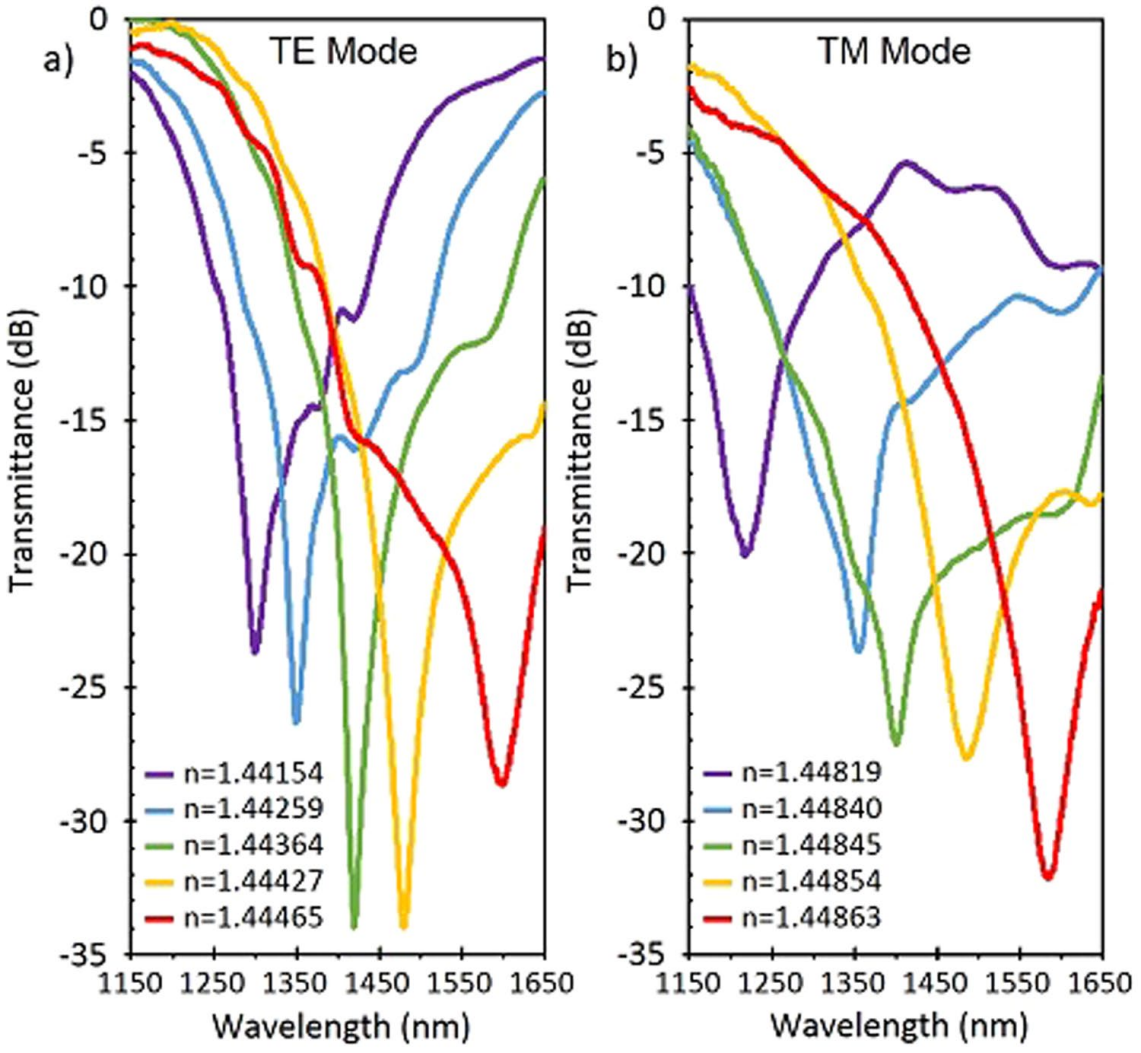
Transmittance spectra obtained when the sensitive region was submerged in solutions of different refractive index. The absorption peaks shift to greater wavelengths when the SMRI increases. (**a**) Spectra corresponding to the TE mode when the fiber is submerged in solutions of 1.4415–1.4447 SMRI. (**b**) Spectra corresponding to the TM mode when the fiber is submerged in solutions of 1.4481–1.4487 SMRI

After submerging the fiber in subsequent solutions of increasing refractive index, the first resonance (LMR_TE_) appears in media of refractive index between 1.4415 and 1.4447 for the spectral range observed (Fig. 2a). As the refractive index of the solution surrounding the fiber increases, the LMR shifts to higher wavelengths. It is worth noting the narrow shape of this resonances. The FWHM obtained for the resonances on the TE Mode is 9 nm. The resonances of the TM mode are slightly wider and their FWHM is 36.5, but this mode is notoriously more sensitive.

As the fiber is submerged in solutions of greater refractive index, the first LMR shifts outside our window. The following resonance is generated in the TM Mode of propagation and it is necessary to change the state of polarization in order to observe it. It should be noted that, using a wider spectral range (not possible with our current configuration setup), both resonances (LMR_TE_ and LMR_TM_) could be observed within certain values of refractive index, but not at the same time. In this last hypothetical case a proper polarization state adjustment is required in order to observe the resonance corresponding to each mode. In our setup, the wavelength range was mainly limited by the single mode fiber the working range of the polarizer, and the OSA.

The following LMR is observed in the surrounding medium refractive index (SMRI) range between 1.4481 and 1.4487 (Fig. 2b). This second resonance (LMR_TM_) shows a greater shift than the previous mode. This also agrees with previous results^11^ that proved that the sensitivity increases for higher SMRI.

The fact that these resonances appear at two separate SMRI ranges should not be a problem when measuring a sample of unknown refractive index. A simple calibration of the polarization state with a reference sample allows to predetermine the working range (the corresponding to the TE or TM mode) of the sensor for every measurement.

It must be noted that the response time of the sensor is shorter than the sweep time of the OSA (below 5 seconds), and no significant variation in the spectrum takes places afterwards. It is also important to note that this test was repeated in different days observing negligible differences in the peak wavelength of the resonances.

**Figure 3:**
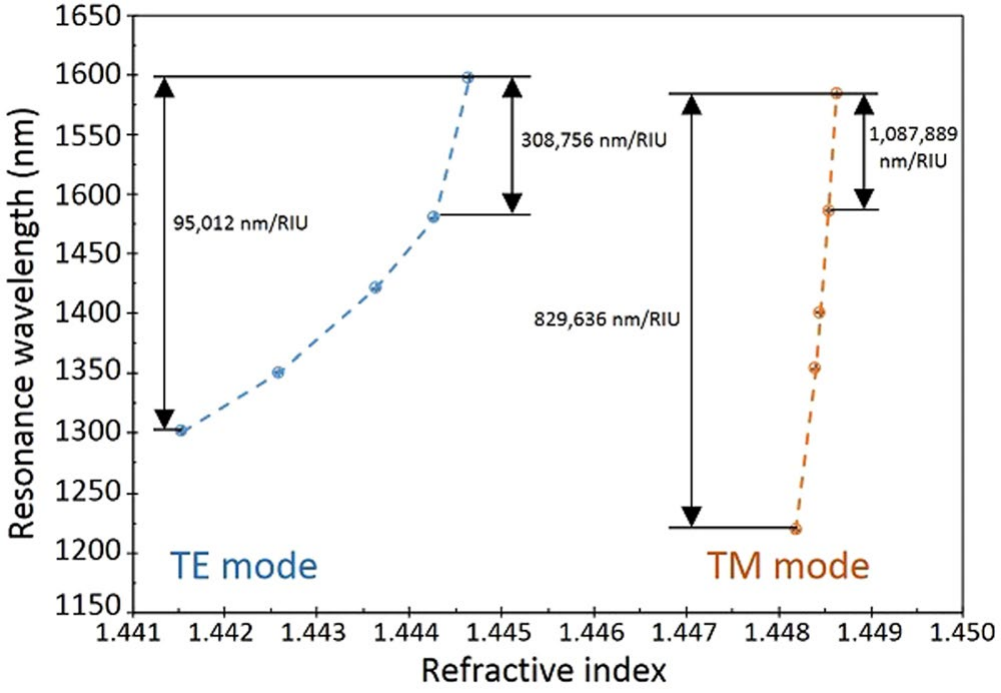
Representation of the central wavelength of the resonances as a function of the refractive index of the surrounding media (SMRI). The slope increases with the SMRI. The error in the Y axis is 0.5 nm, and in the X axis it is 0.000005.

The central wavelength of each LMR as a function of the refractive index of the solutions is shown in Fig. 3 for a more meaningful representation of the sensitivity of the devices. Here, we can observe that each mode shows different sensitivity, higher sensitivity for higher SMRI as it was mentioned before. In can also be seen that the sensitivity curves obtained for TE and TM modes are not linear, as sensitivity increases for higher SMRI. In particular, the LMR_TE_ obtains an average sensitivity of 95,012 nm/RIU, but its highest sensitivity is 308,756 nm/RIU. This figure overpasses the sensitivity obtained in previous works for similar sensors fabricated with ITO thin films^11^.

For higher refractive index, the LMR_TM_ shows even greater sensitivity. Although it is true that the different modes might lead to resonances with different sensitivities, the main responsible for the increment in sensitivity in this case is the increment in the SMRI^7^. LMR_TM_ seems to show a linear behaviour but this can be associated to the small refractive index variations between each point with an average sensitivity of 829,636 nm/RIU. For the highest SMRI range, LMR_TM_ shows a record sensitivity of 1,087,889 nm/RIU. This achievement means that using an OSA with a standard resolution of one picometer, a variation of 9.19 × 10^−10^ units of refractive index could be achieved. In order to compare this figures, we can calculate the figure of merit (FOM) as the ratio sensitivity/FWHM, for the LMR_TE_ we would obtain a value of 10,556.89 RIU^−1^. Although the LMR_TM_ is wider, it has a higher sensitivity and, in its most sensitive region, the FOM is 29,805.18 RIU^−1^. To our knowledge, there is no experimental report of any optical device based on lossy mode resonances capable of such sensitivity and FOM.

## Conclusions

A lossy mode resonance based optical fiber sensor was successfully fabricated. The precise control of the tin dioxide thin-film coating the D-Shaped fiber and the use of polarized light has allowed to obtain a record sensitivity of 1,087,889 nm/RIU. High sensitivity refractometers based on this configuration had been presented before. Unlike, here we show how the optimal choice of materials and the thickness of the film can determine the sensitivity and performance of these sensors. Particularly, we have presented here a refractometer 3.5 times more sensitive than those reported before^11^ using the same technique.

The operational range between 1.4601 and 1.4607 suggests the use in industrial applications such as gear lubricant oil degradation. Since it is also possible to tune the resonances in another range by modifying the thickness of the coating, it can also be used for other purposes such as the determination of degrees °Brix in a solution, for biomedical or gas sensing applications where other sensors do not offer enough sensitivity for the detection of indicators or optical fiber arrangement can be advantageous. Although here we focus on the sensibility to bulk modifications on refractive index, it has already been reported in previous works of our group how these devices can be functionalized to work as surface detectors of proteins^22^. These devices can take advantage of the improvement of sensibility and optimization reported here. The sensitivity obtained in this research is a huge improvement over the sensors studied previously and can lead to the development of new sensors with the difficulties of positioning the resonance within the working range of the detection equipment as it was mentioned above. This achievement opens the possibility to obtain levels of detection that could not be obtained before, which could even be improved by the use of other materials, or metamaterials whose optical properties are specifically designed to maximize the sensitivity.

It is also worth noting that, due to the fact that the observed resonances are narrow, it would be possible to multiplexing several sensors in wavelength, obtaining all the benefits that this kind of configuration offers. Although SPR based devices are currently more popular, LMR based sensors have already proved to be a competent technology, featuring characteristics that overcome those of the SPR. In particular, the possibility to use a wide range of materials (metallic oxides or polymers, for example) or the high sensibility observed in this paper may be the key for this technology to succeed. The results presented here show that LMR based technology offers a wide range of possibilities and may become a standard for resonance based sensors in the early future.

## Materials and Methods

D-shaped fiber is a Standard Single Mode Fiber (Corning SMF-28) with a polished length of 1.7 cm obtained from Phoenix Photonics LTD. This fiber has an attenuation peak of 3 dB at 1550 nm in high refractive index oil characterizing the polishing process. A higher attenuation corresponds to a lower distance between polished side and core of the fiber although, according to previous studies, this parameter does not affect to the sensitivity of the resonances, but to their shape^11^.

Tin dioxide thin-film (SnO_2_ 99.99% sputtering target from Zhongnuo Advanced Materials) was deposited onto a D-Shaped single mode optical fiber using a DC sputter coater (Emitech K675X from Quorum Technologies). The conditions of fabrication in the sputter coater were 9 × 10^−2^ mbar of pressure in an argon atmosphere with 90 mA of current. Two different setups were used during the deposition: one for the study of the films and the resonances for various thicknesses (fabrication set-up), and the second to characterize the device as a refractometer (refractometer characterization setup).

### Fabrication setup

**Figure 4:**
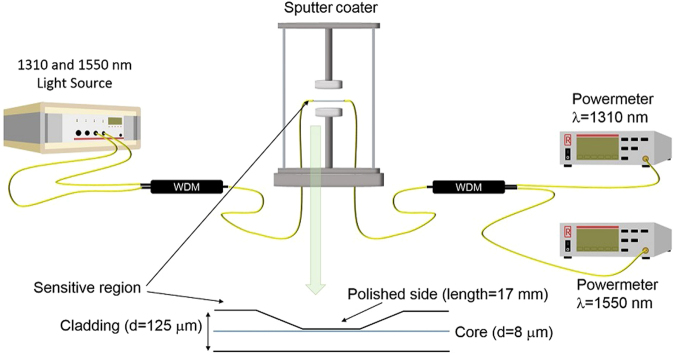
Fabrication setup used for monitoring the output at different wavelengths while the deposition of the SnO_2_ thin film. The sensitive region is a 17 mm side-polished segment of a standard single mode fiber, located horizontally under the target inside the sputter coater.

A couple of laser sources (BCP 400 A) at two wavelengths (1310 and 1550 nm) were used for this setup as shown on Fig. 4. The output signal was measured with power meters (RIFOCS 575 L) connected to a data logger (HP 34970 A). In order to couple the light at both wavelengths, a set of two wavelength division multiplexers (WDM) from Telnet Redes Inteligentes Inc. were used.

### Refractometer characterization setup

**Figure 5:**
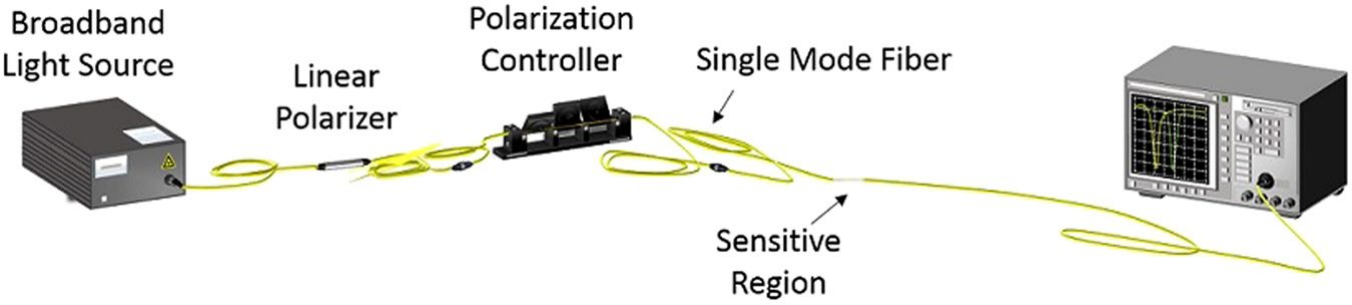
Setup used for the characterization of the sensor as refractometer. It allows controlling the state of polarization of the light guided through the sensitive region and monitor the output spectrum.

The setup shown on Fig. 5 was used for the refractometer characterization. Here, the light from a broadband super luminescent emitting diode (HP 83437 A) source goes through an in-fiber linear polarizer (Phoenix Photonics), then through a manual polarization controller (Thorlabs FPC032). The output of the polarization controller is connected to the SnO_2_ coated side-polished fiber. Finally, the output of the D-shape fiber is connected to an Optical Spectrum Analyzer (HP 86142 A) where data is collected. The resulting spectra were then processed with an algorithm in Matlab in order to obtain the corresponding peak wavelengths.

In order to perform the measurements, the coated region was sequentially submerged in several Glycerin solutions (Panreac Technical Grade) with different refractive indexes. Since the device shows a high sensitivity, it is necessary to obtain several solutions in which the refractive index has really small variations. All the solutions were carefully prepared and stirred for several hours. Then, their refractive index was measured with a commercial refractometer (Mettler Toledo Refracto 30GS) 10 times and averaged in order to minimize the error.

## Electronic supplementary material

Supplementary Information
