# Supplementary material for: Is there a frontier in sensitivity with Lossy mode resonance (LMR) based refractometers?
Source: Sci Rep. 2017 Aug 31;7:10280. doi: 10.1038/s41598-017-11145-9 (PMC5579297; doi:10.1038/s41598-017-11145-9)
Supplement: Supplementary file 1 — Supplementary Information [file 41598_2017_11145_MOESM1_ESM.pdf]

Is there a frontier in sensitivity with Lossy mode resonance (LMR) based refractometers?

Aritz Ozcariz[1,2,*], Carlos R. Zamarreño[1,2], Pablo Zubiate[1,2], Francisco J. Arregui[1,2]

[1]Department of Electrical and Electronic Engineering, Public University of Navarre, 31006 Pamplona, Spain
[2]Institute of Smart Cities, Public University of Navarre, 31006 Pamplona, Spain
*aritz.ozcariz@unavarra.es

Supplementary Information

<table>
<tr><th>time (minutes)</th><th>thickness (nm)</th></tr>
<tr><td rowspan="6">2</td><td>93,81</td></tr>
<tr><td>89,11</td></tr>
<tr><td>92,29</td></tr>
<tr><td>93,9</td></tr>
<tr><td>90,82</td></tr>
<tr><td>91,52</td></tr>
<tr><td rowspan="4">3</td><td>128</td></tr>
<tr><td>137,5</td></tr>
<tr><td>131,2</td></tr>
<tr><td>135,4</td></tr>
<tr><td rowspan="5">4</td><td>182,8</td></tr>
<tr><td>200,1</td></tr>
<tr><td>207,3</td></tr>
<tr><td>200,1</td></tr>
<tr><td>200,4</td></tr>
</table>

Supplementary table S1. Measurements of the thickness of several coatings of SnO_2 sputtered for 2, 3 and 4 minutes. This data has been used to calculate the average deposition rate of the coatings.

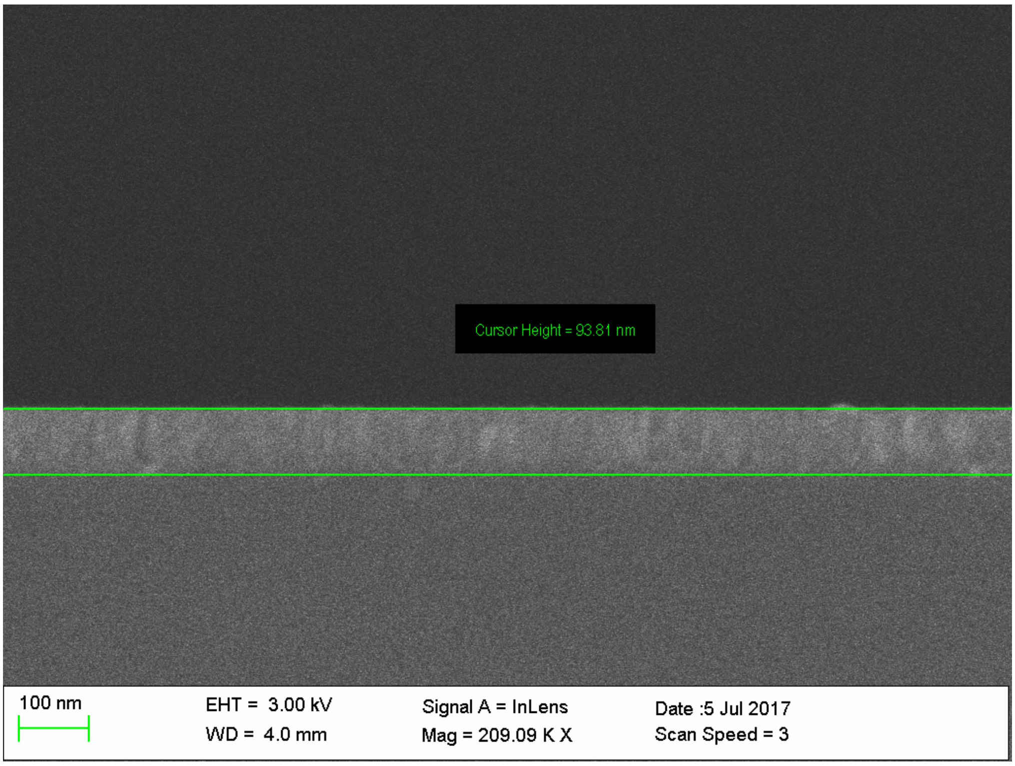

Supplementary figure S1. SEM image on a transversal cut of a SnO_2 coating sputtered on silicon wafer for 2 minutes.

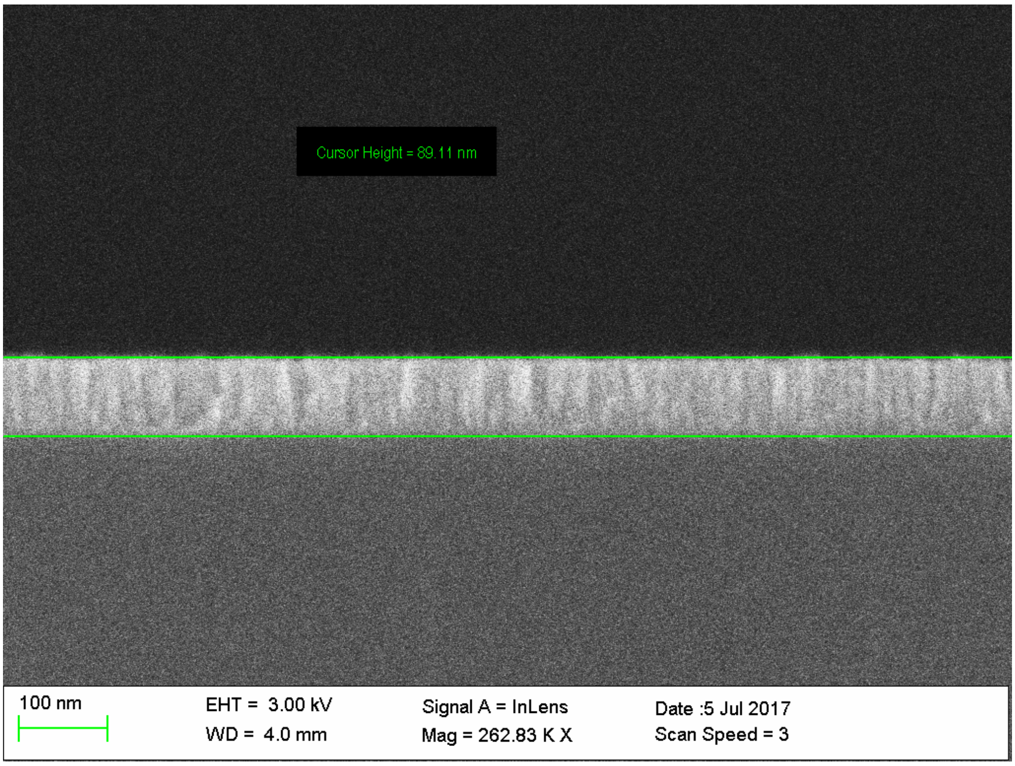

Supplementary figure S2. SEM image on a transversal cut of a SnO_2 coating sputtered on silicon wafer for 2 minutes.

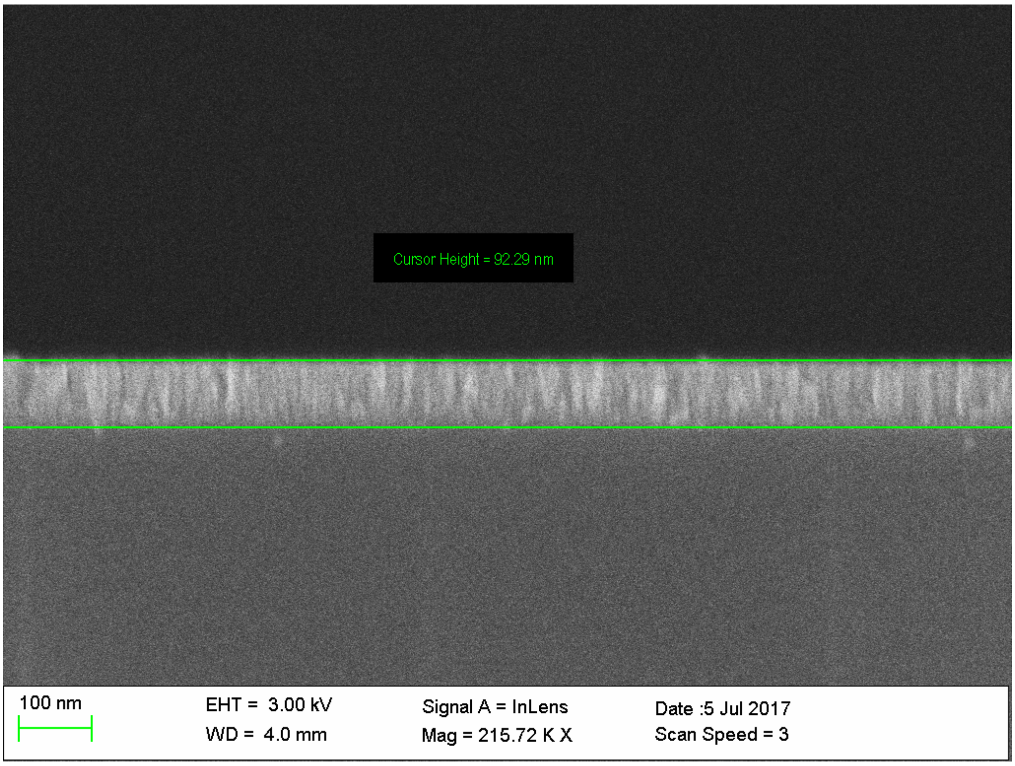

Supplementary figure S3. SEM image on a transversal cut of a SnO_2 coating sputtered on silicon wafer for 2 minutes.

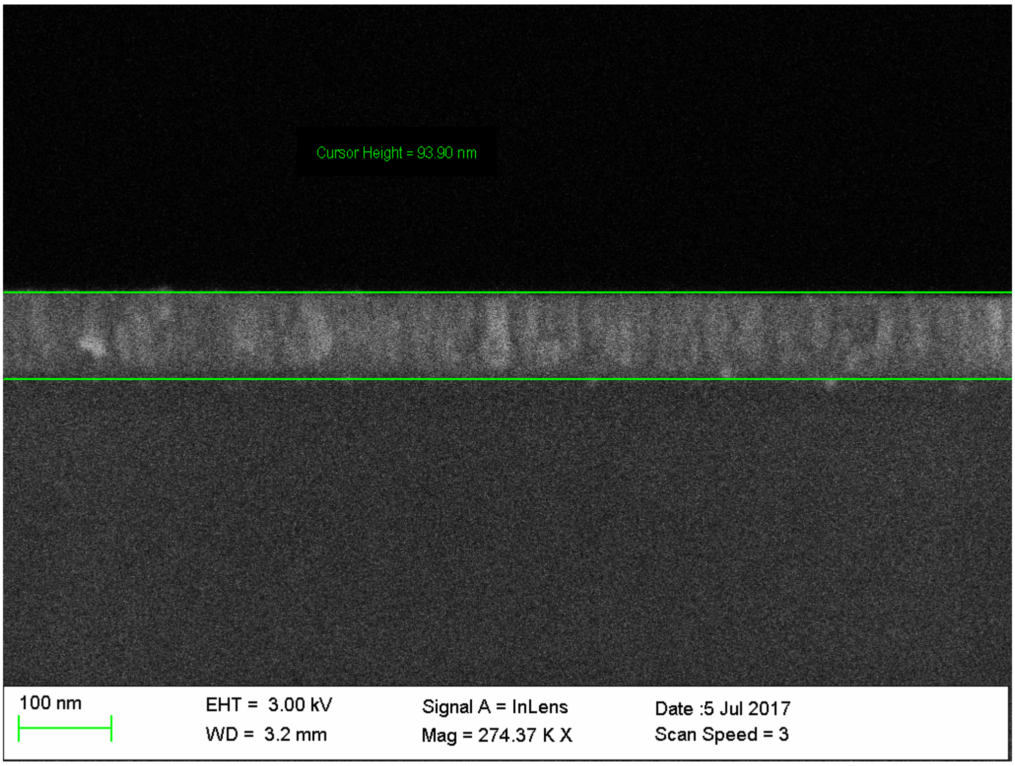

Supplementary figure S4. SEM image on a transversal cut of a SnO_2 coating sputtered on silicon wafer for 2 minutes.

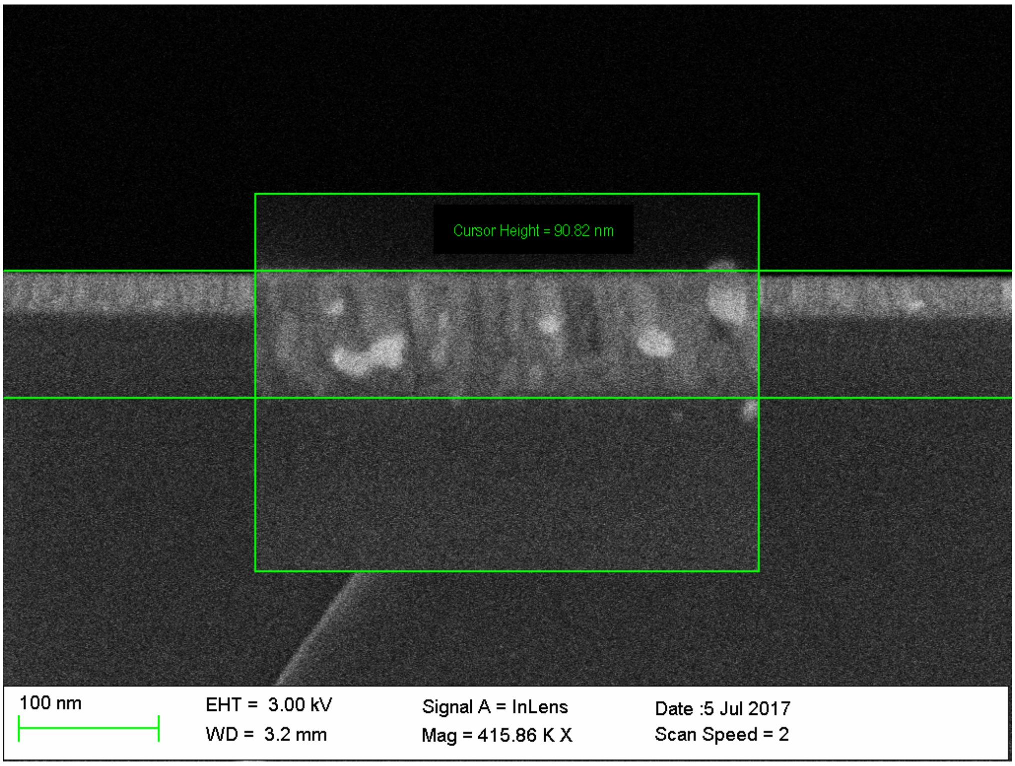

Supplementary figure S5. SEM image on a transversal cut of a SnO_2 coating sputtered on silicon wafer for 2 minutes.

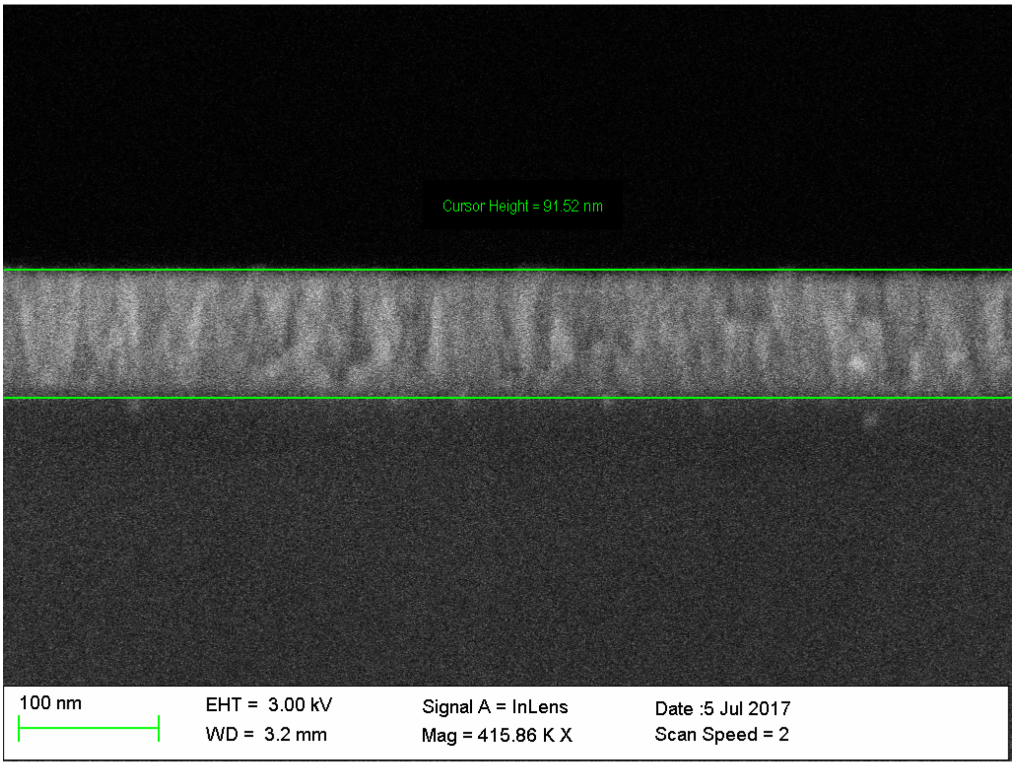

Supplementary figure S6. SEM image on a transversal cut of a SnO_2 coating sputtered on silicon wafer for 2 minutes.

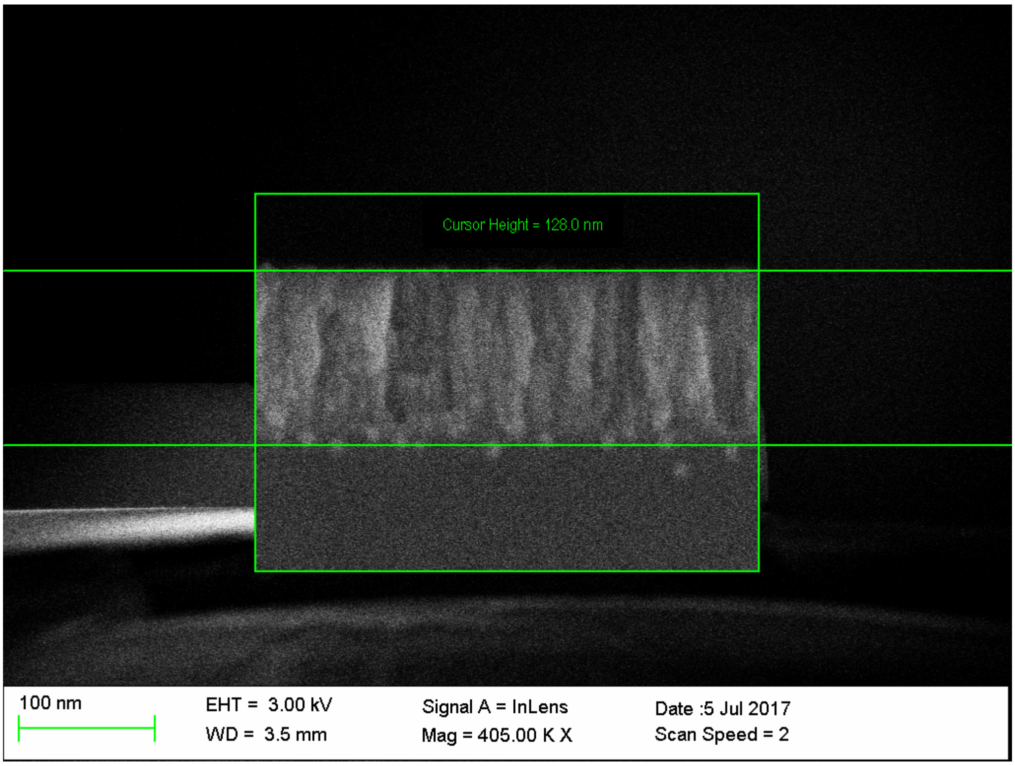

Supplementary figure S7. SEM image on a transversal cut of a SnO_2 coating sputtered on silicon wafer for 3 minutes.

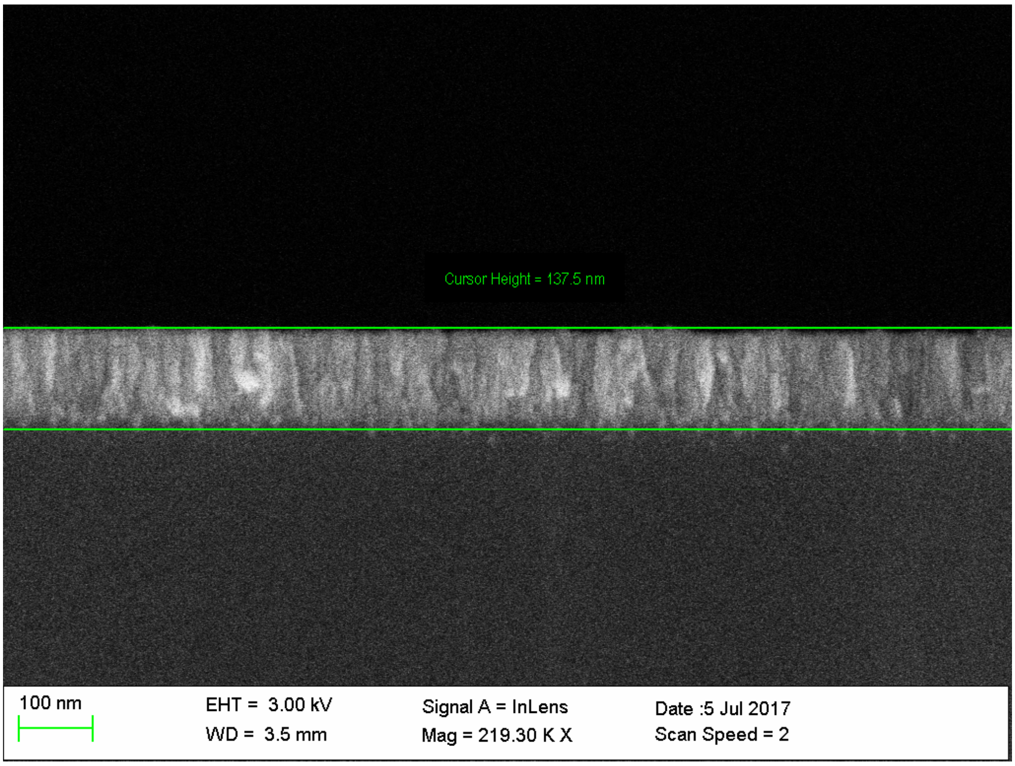

Supplementary figure S8. SEM image on a transversal cut of a SnO_2 coating sputtered on silicon wafer for 3 minutes.

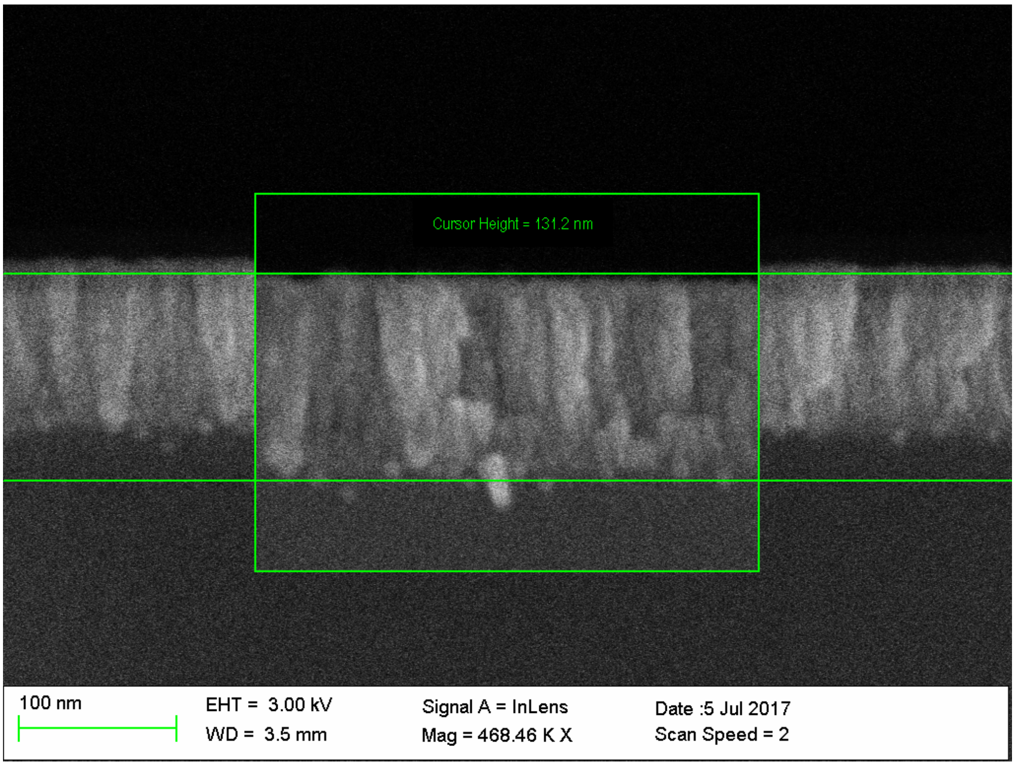

Supplementary figure S9. SEM image on a transversal cut of a SnO_2 coating sputtered on silicon wafer for 3 minutes.

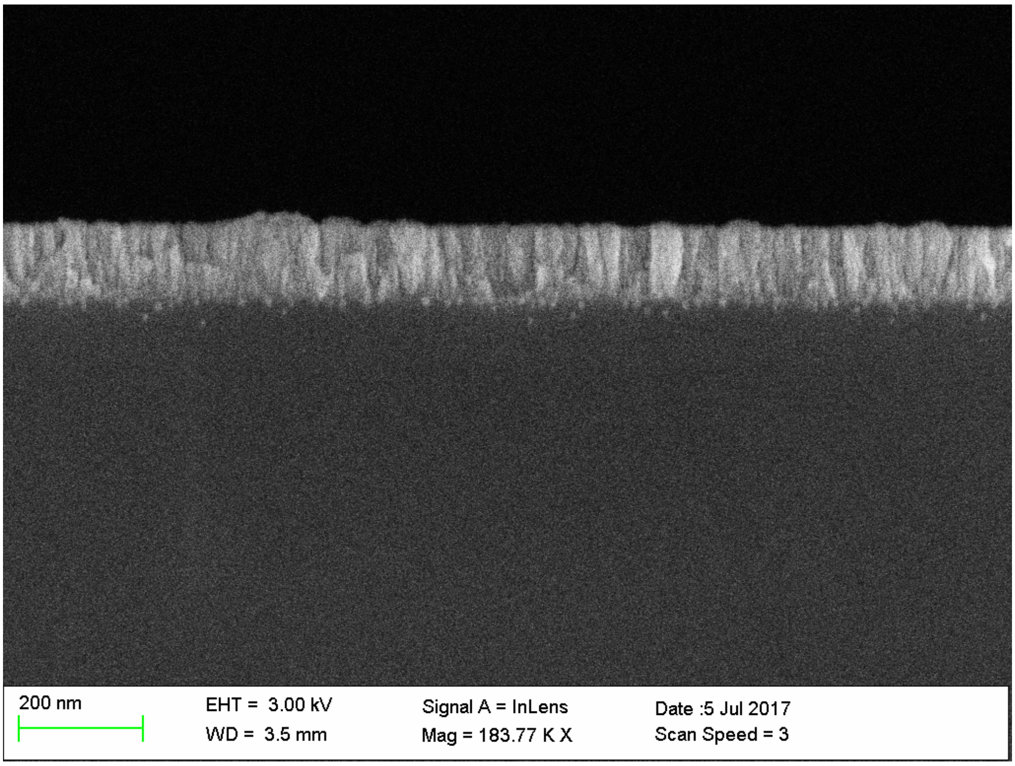

Supplementary figure S10. SEM image on a transversal cut of a SnO_2 coating sputtered on silicon wafer for 3 minutes.

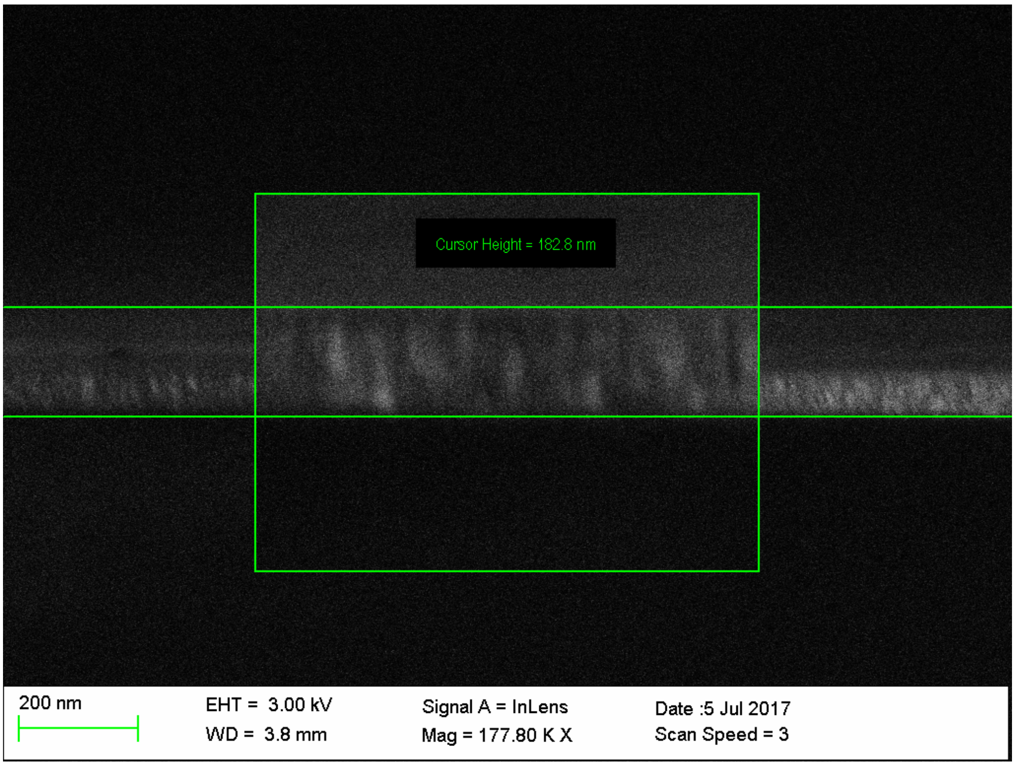

Supplementary figure S11. SEM image on a transversal cut of a SnO_2 coating sputtered on silicon wafer for 4 minutes.

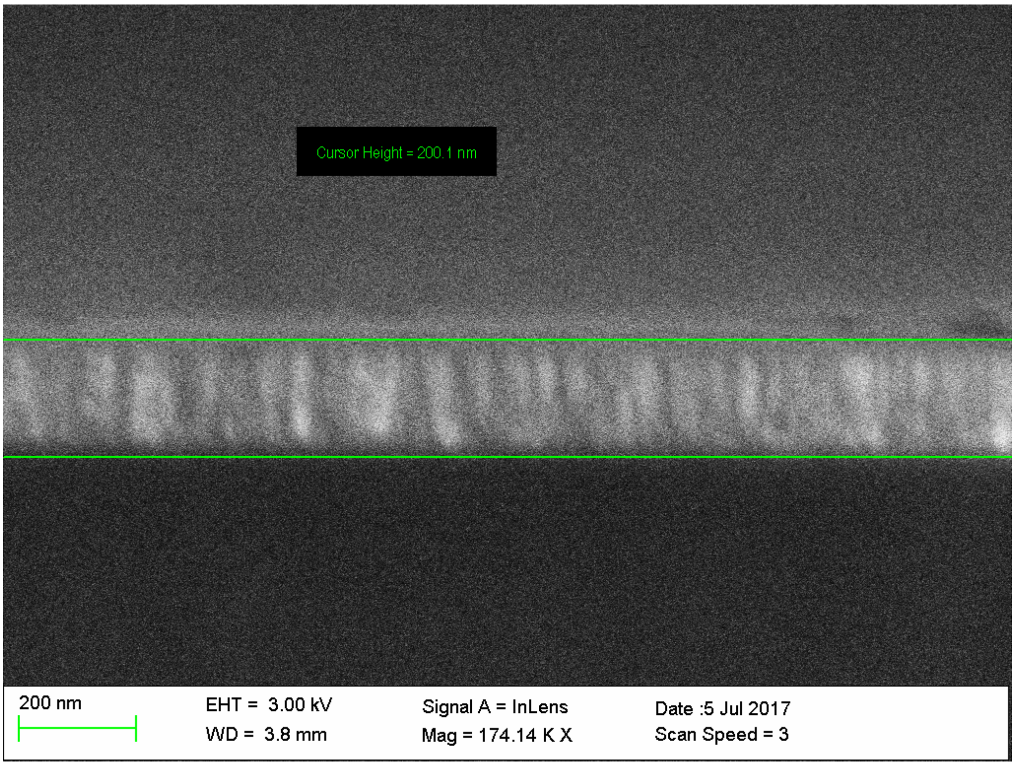

Supplementary figure S12. SEM image on a transversal cut of a SnO_2 coating sputtered on silicon wafer for 4 minutes.

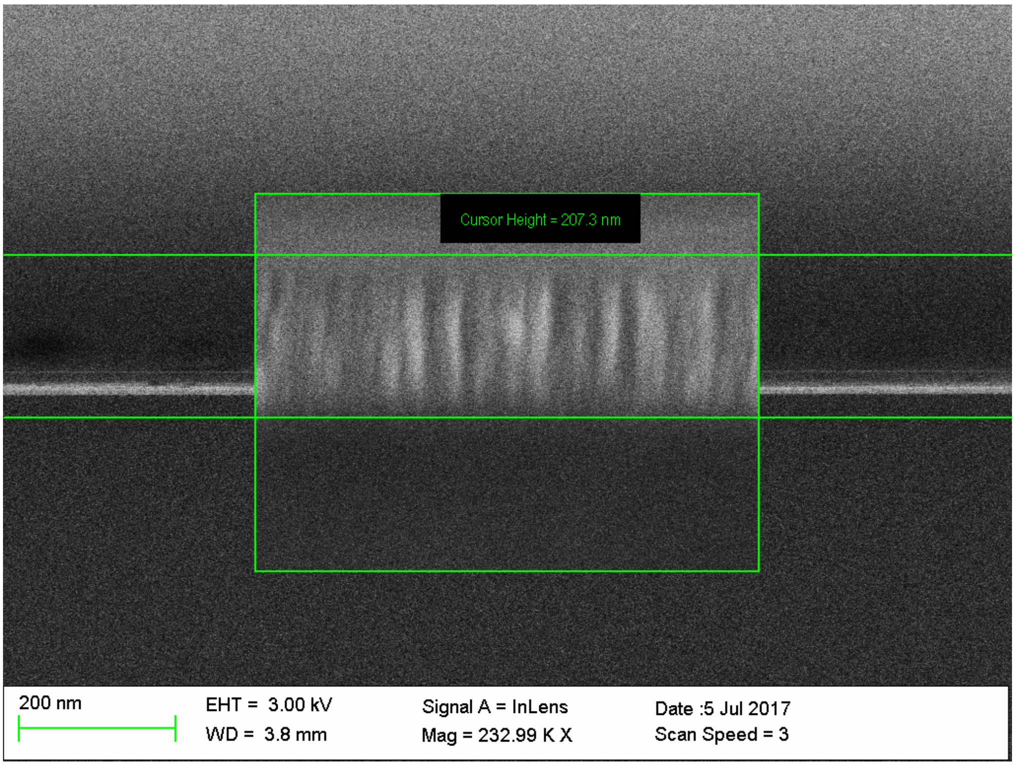

Supplementary figure S13. SEM image on a transversal cut of a SnO_2 coating sputtered on silicon wafer for 4 minutes.

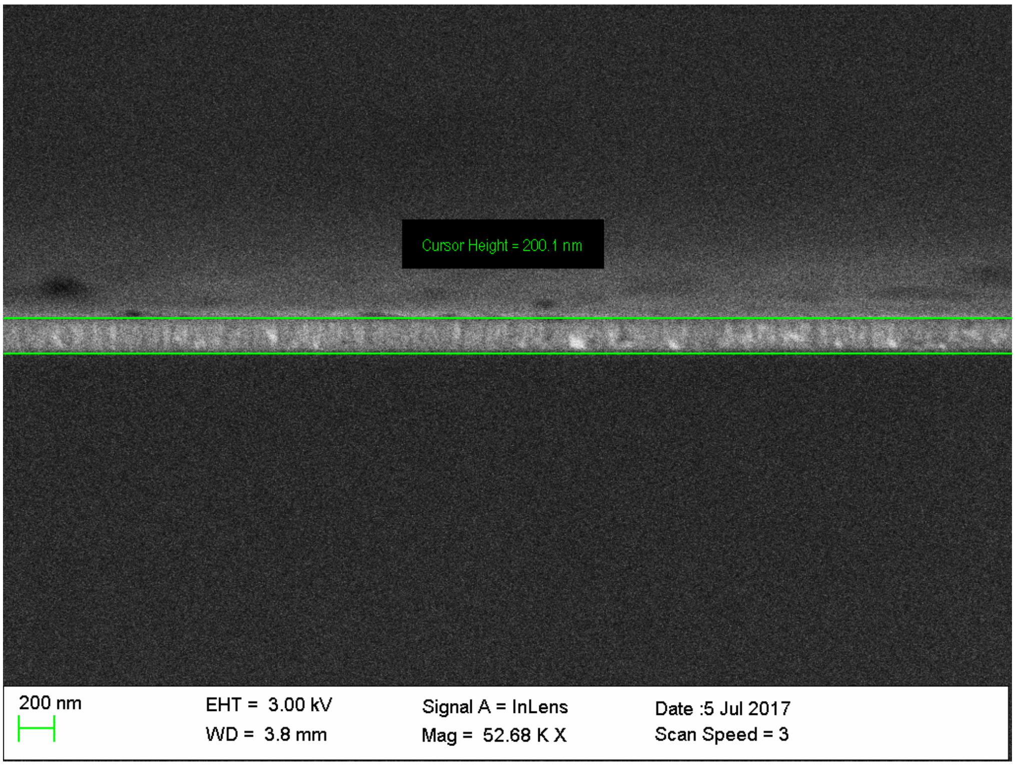

Supplementary figure S14. SEM image on a transversal cut of a SnO_2 coating sputtered on silicon wafer for 4 minutes.

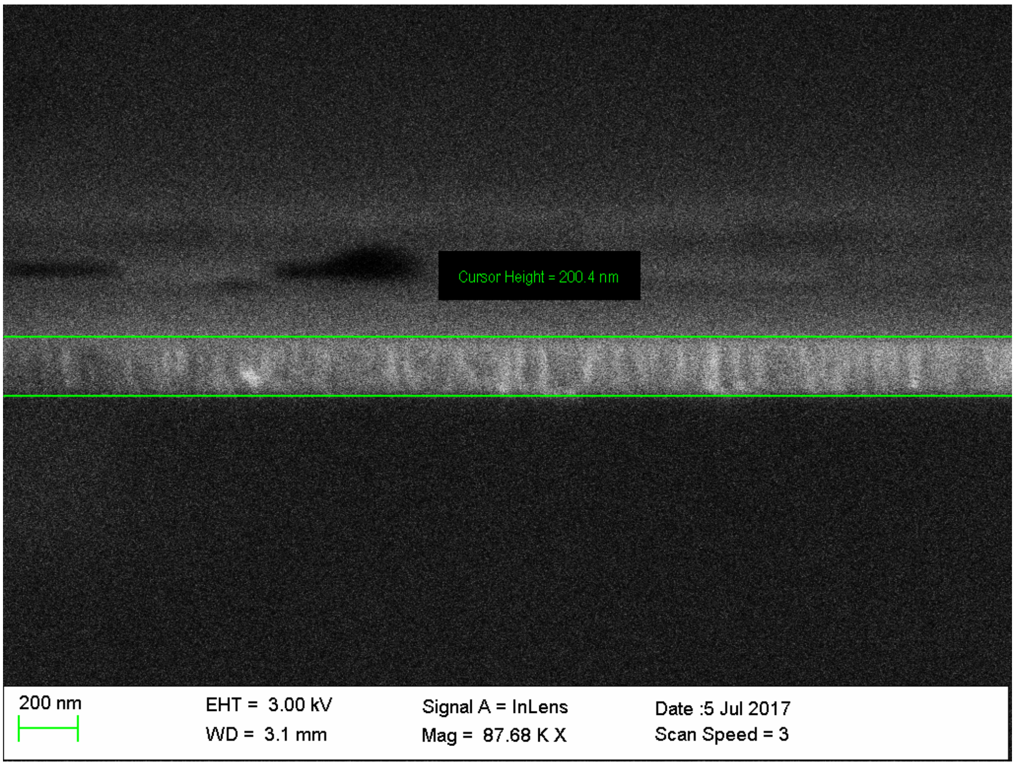

Supplementary figure S15. SEM image on a transversal cut of a SnO_2 coating sputtered on silicon wafer for 4 minutes.

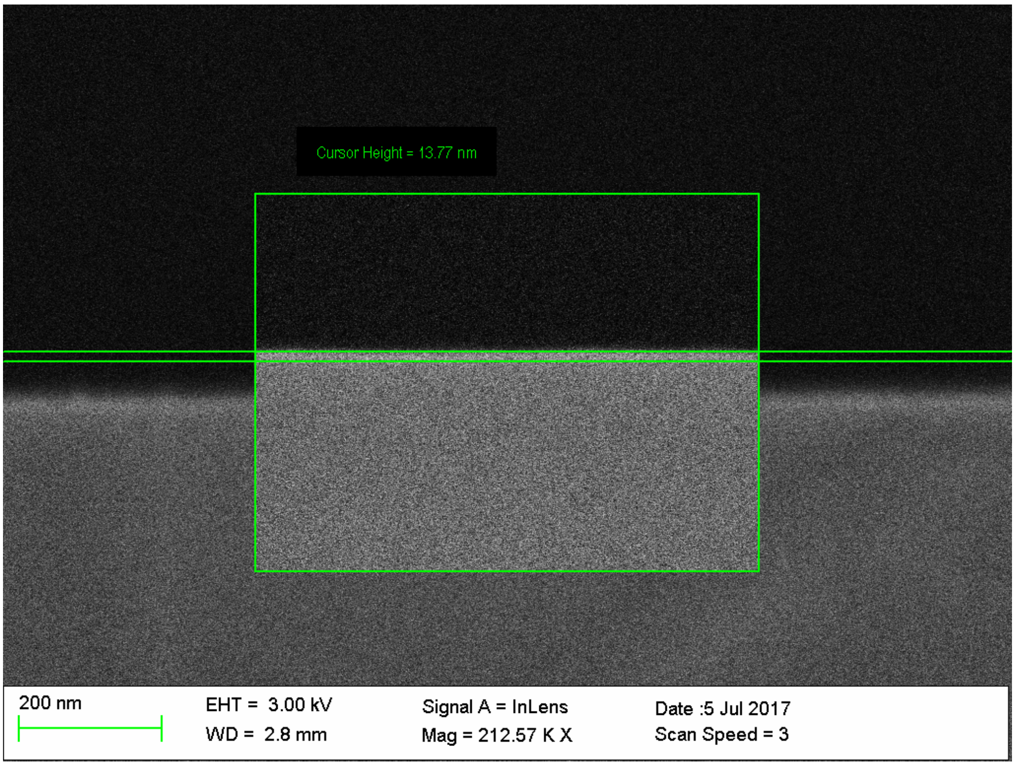

Supplementary figure S16. SEM image on a transversal cut of a 13.44 nm SnO_2 coating sputtered on silicon wafer for 20 seconds. This coating corresponds to the one of the sensor described on the paper.
